# Recent Advances in the Synthesis of 2-Pyrones

**DOI:** 10.3390/md13031581

**Published:** 2015-03-23

**Authors:** Jong Seok Lee

**Affiliations:** 1 Marine Natural Products Chemistry Laboratory, Korea Institute of Ocean Science and Technology (KIOST), Ansan 426-744, Korea; 2 Department of Marine Biotechnology, Korea University of Science and Technology, Daejeon 305-350, Korea; E-Mail: jslee@kiost.ac.kr; Tel.: +82-31-400-6173; Fax: +82-31-400-6170

**Keywords:** 2-pyrone, synthesis, marine natural product, metal-catalyzed reaction

## Abstract

The present review summarizes the recent progresses in the synthesis of 2-pyrones and the application to the synthesis of marine natural products. Especially, much attention was placed on the transition metal catalyzed synthetic methodologies in this review.

## 1. Introduction

**Figure 1:**
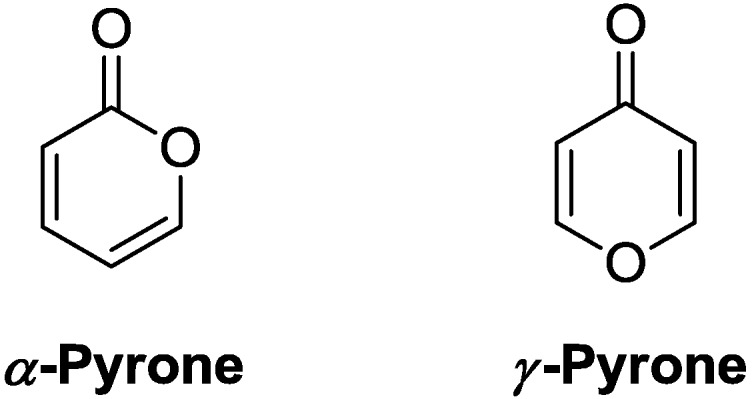
Structures of pyrones.

Pyrones constitute a family of six-membered unsaturated cyclic compounds containing an oxygen atom. In view of chemical motifs, γ-pyrone is the vinylogous form of α-pyrone (instead of α-pyrone, the term “2-pyrone” will be used hereafter), which possesses a lactone. As a result, these ring systems share similar chemical properties (Figure 1) [1,2,3].

**Figure 2:**
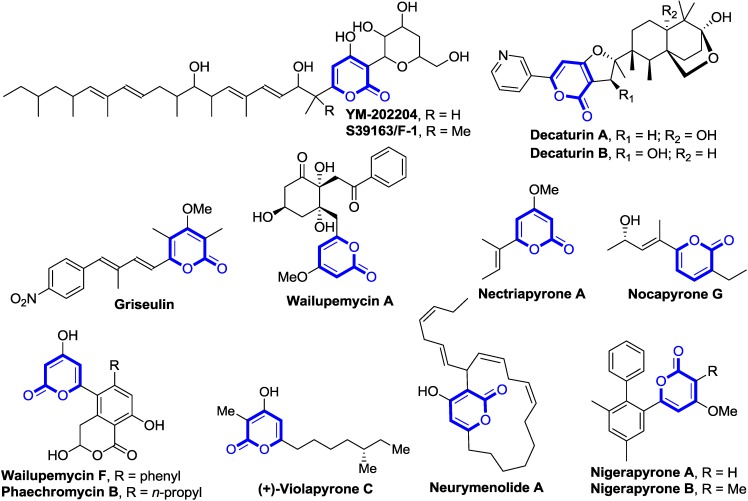
Representative marine natural products bearing a 2-pyrone motif [5,6,7,8,9,10,11,12,13,14,15,16,17,18].

2-pyrone is extremely prevalent in numerous natural products isolated from plants, animals, marine organisms, bacteria, fungi, and insects that exhibit a broad range of biological activities, such as antifungal, antibiotic, cytotoxic, neurotoxic and phytotoxic(Figure 2) [4]. Moreover, 2-pyrone can serve as a versatile building block for the synthesis of key intermediates in synthetic organic chemistry as well as in medicinal chemistry due to the existence of functional groups, such as conjugated diene and the ester group. Thus, development of a highly efficient synthetic method affording substituted 2-pyrones under mild conditions has been one of considerable attention in organic chemistry.

For all its importance in organic chemistry and medicinal chemistry, it was not until recently that various transition metal-catalyzed methods for the efficient synthesis of 2-pyrone have been developed and thus we were able to have a clear understanding of the exact nature and the unique chemical behavior of 2-pyrone. The present review will be concerned with the recent advances in the synthesis of 2-pyrones with a special emphasis on metal-catalyzed methods, but other significant methods will be covered as well as the synthesis of complex marine natural products.

## 2. Synthesis of 2-Pyrones

### 2.1. Metal-Catalyzed Syntheses

#### 2.1.1. Palladium-Catalyzed Synthesis of 2-Pyrones

**Scheme 1:**
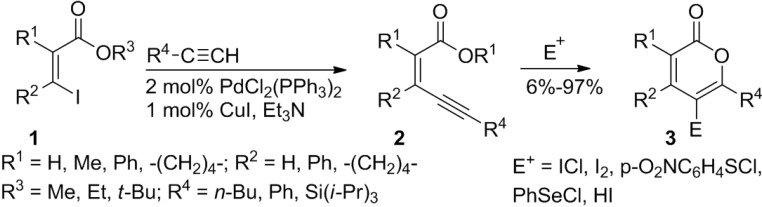
A consecutive two-step approach to isocoumarins and 2-pyrones involving Sonogashira coupling reaction.

Larock and co-workers demonstrated a consecutive two-step approach to 2-pyrones employing a Sonogashira coupling reaction to prepare (*Z*)-2-alken-4-ynoates, followed by electrophilic cyclization (Scheme 1) [19]. Although in some cases this protocol affords 5-membered lactones or mixtures of 5- and 6-membered lactones, it is compatible with various alkynyl esters bearing diverse functional groups and readily provides the anticipated 2-pyrones. Further treatment of the products with electrophiles such as ICl, I_2_, PhSeCl, p-O_2_NC_6_H_4_SCl, and HI affords the corresponding substituted 2-pyrones. Especially, iodo-2-pyrone would be a key intermediate in synthesis of more complex molecules (Scheme 1).

**Scheme 2:**
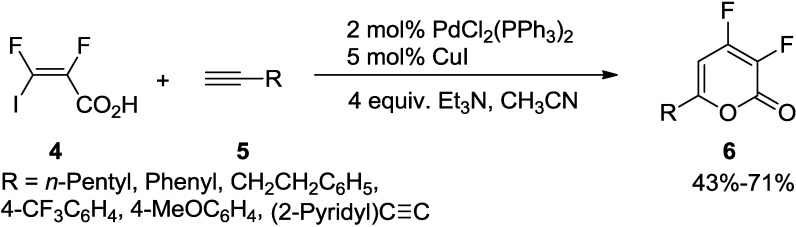
Synthesis of difluorinated 2-pyrones involving Sonogashira alkynylation.

Similarly, Burton and co-workers developed an efficient method for the synthesis of difluorinated 2-pyrones [20]. This synthetic protocol involves a reaction of (2*E*)-2,3-difluoro-3-iodoacrylic acid (**4**) with diverse terminal acetylenes (**5**) under the Sonogashira alkynylation condition using PdCl_2_(PPh_3_)_2_ in combination with CuI as a co-catalyst to furnish difluorinated 2-pyrones (**6**) as the sole product in good yields (Scheme 2).

**Figure 3:**
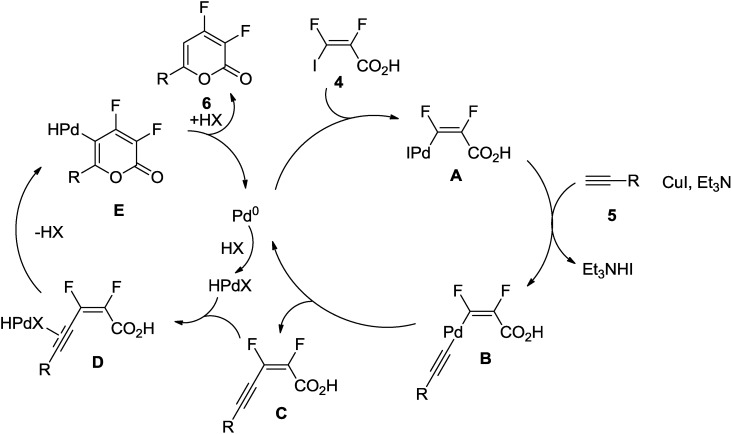
Proposed Mechanism.

The proposed catalytic cycle suggests that the iodoacid (**4**) reacts with the alkyne in the presence of Pd(0) to produce the enynoic acid (**C**) (Figure 3). Regenerated Pd(0) species in the first cycle is again oxidized into Pd(II) by an acid moiety HX (X could be I-, Cl-) in the reaction. In the second catalytic cycle, 2-pyrone is formed by the cyclization of the enynoic acid under the catalysis of Pd(II), followed by reductive elimination generating the Pd(0).

**Scheme 3:**
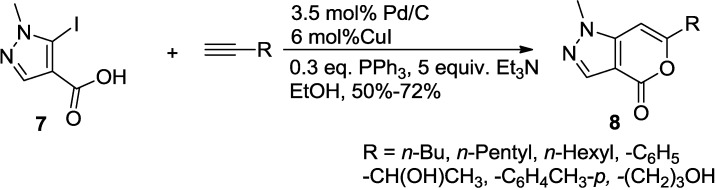
Synthesis of 2-pyrone fused with a pyrazol moiety.

Pal and co-worker further extended the scope of the coupling-cyclization strategy to the regioselective synthesis of 2-pyrone fused with a pyrazol moiety (**8**) (Scheme 3) [21]. This method proceeds via Pd/C-mediated tandem C-C and C-O bond formation between the 5-iodopyrazole-4-carboxylic acid (**7**) and a terminal alkyne to afford pyrano[4,3-*c*]pyrazol-4(1*H*)-one (**8**) in a single pot.

**Scheme 4:**
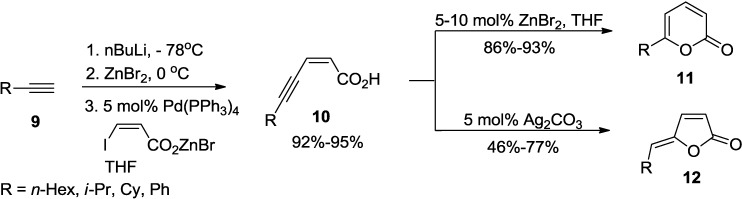
A two-step synthesis of 6-alkyl-2-pyrones employing ZnBr_2_-catalyzed lactonization.

A two-step synthesis of 6-alkyl-2-pyrones recently developed by Negishi and co-workers involves Pd-catalyzed alkynylzinc-haloacrylic acid coupling and ZnBr_2_-catalyzed lactonization (Scheme 4) [22]. Therefore, under this reaction condition, (*Z*)-5-alkyl-2-en-4-ynoic acids (**10**) furnishes 6-alkyl-2H-pyran-2-ones (**11**) in high yields. Although this method provides (*Z*)-5-alkylidenefuran-2(5H)-ones (**12**) as minor products, the ratios of pyranone and furanone are often very high. In contrast, lactonization of **10** catalyzed by Ag_2_CO_3_ provides (*Z*)-5-alkylidenefuran-2(5H)-ones (**12**) selectively in >90% yields along with minor amounts of **11**.

**Scheme 5:**
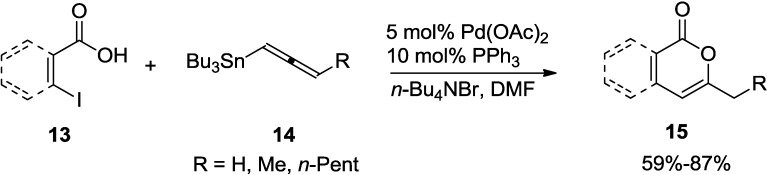
Synthesis of 2-pyrones using allenyl-tributyltin reagents.

As a new addition to the coupling-lactonization strategy, a general route using allenyl-tributyltin reagents (**14**) was developed by Abarbri and co-workers (Scheme 5) [23]. In the reaction, treatment of (*Z*)-β-iodovinylic acids (**13**) or 2-iodobenzoic acids with various allenyl-tributyltin reagents in the presence of palladium acetate, triphenylphosphine, and tetrabutylammonium bromide in dimethylformamide provides to 2-pyrones and 3-substituted isocoumarins (**15**) provided good yields of the corresponding 2-pyrones or 3-substituted isocoumarins via tandem Stille reaction and 6-*endo*-dig oxacyclization.

**Figure 4:**
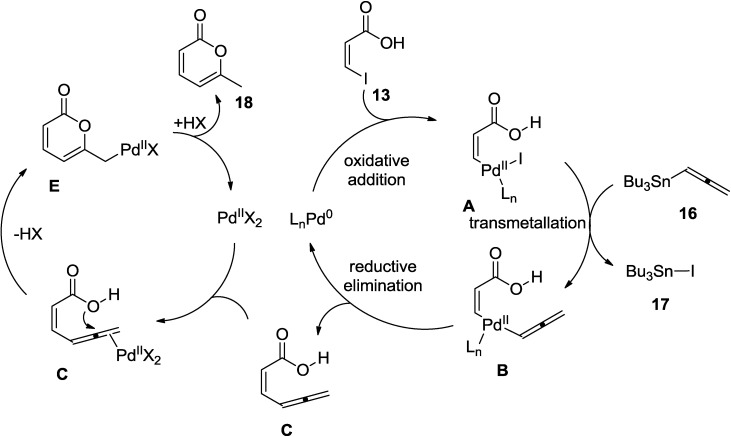
Proposed mechanism.

The actual catalyst in this process is a Pd(0) complex, which is generated by the reduction of Pd(OAc)_2_ (Figure 4). In the proposed mechanism, the vinylic (or aromatic) iodide (**13**) is added to Pd(0) oxidatively and the allenyl-tributyltin (**16**) reagent would be cross-coupled with the vinylic iodide (**13**) to afford the corresponding 3-allenylpropenoic acid (**C**) by transmetallation and reductive elimination. The allene moiety is further activated by complexation with Pd(II) and undergoes intramolecular 6-*exo*-dig nucleophilic attack by the carboxylic group. At the end of the catalytic cycle, 2-pyrone (**18**) is formed while Pd(II) is regenerated to enter into a new reaction cycle.

**Scheme 6:**
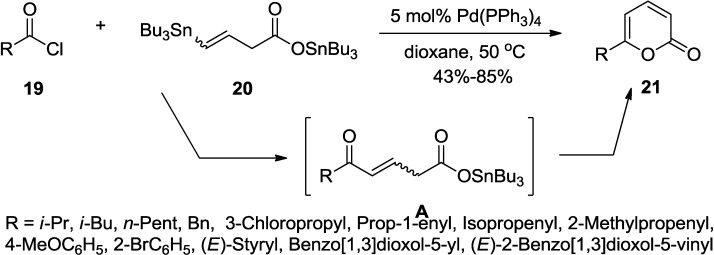
Synthesis of 2-pyrones using tributylstannyl 5-substituted 5-oxopent-3-enoate.

2-Pyrone synthesis by a direct coupling of a functional vinylstannane to an acyl chloride is an intriguing example developed by Parrain and co-workers (Scheme 6) [24]. This annulation most probably proceeds via a Stille reaction/cyclization sequence in good yields.

The tributylstannyl 5-substituted 5-oxopent-3-enoate (**A**) would be formed in the first step through consecutive transformations of oxidative addition, transmetallation, and reductive elimination. It is proposed that the dienol derived from (**A**) might be the key player in the lactonization reaction of the tributylstannyl 5-substituted 5-oxopent-3-enoate (**A**).

**Scheme 7:**
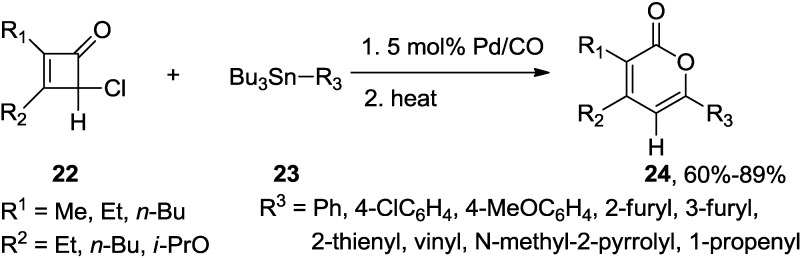
Synthesis of 2,3,6-trisubstituted-2-pyrones involving a palladium-catalyzed carbonylative cross-coupling.

Liebeskind and co-workers reported a general method for the synthesis of 2,3,6-trisubstituted-2-pyrones (**24**), involving a palladium-catalyzed carbonylative cross-coupling of 4-chloro-2,3-disubstituted-2-cyclobutenones (**22**) with alkenyl-, aryl-, and heteroaryltin reagents (**23**) and thermolysis (Scheme 7) [25]. This regiospecific reaction has a position preference to the 4-position of the cyclobutenone in the cross-coupling step.

**Figure 5:**
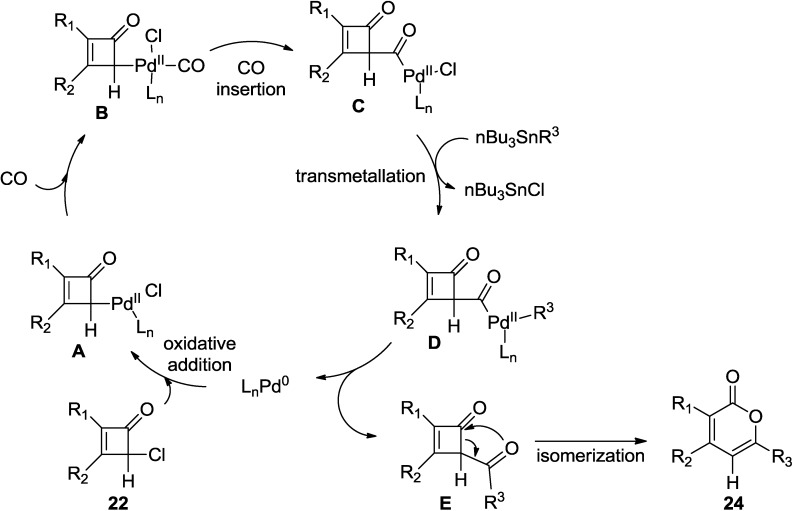
Proposed mechanism.

Mechanistically, this reaction follows Stille-carbonylative cross-coupling reaction (Figure 5). Thus, the initial step involves the oxidative addition of 2-cyclobutenones (**22**) to the palladium catalyst to form a palladium complex (**A**). Subsequently, CO insertion of **B** provides **C**, which further undergoes transmetallation with nBu_3_Sn-R^3^ to deliver 4-acyl-2-cylobutenone (**E**). Interestingly, this transient intermediate rapidly isomerizes to 2-pyrones (**24**).

**Scheme 8:**
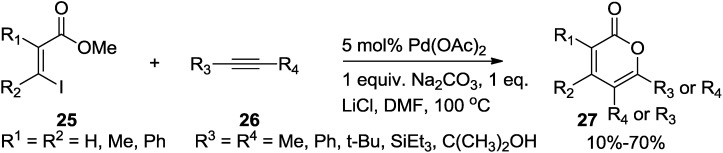
Pd-catalyzed annulation reaction involving α,β-unsaturated esters with internal alkynes.

Larock and co-workers developed an especially simple and convenient, regioselective route to isocoumarins and 2-pyrones containing aryl, silyl, ester, tert-alkyl, and other hindered groups (Scheme 8) [26]. Treatment of halogen- or triflate-containing aromatic and α,β-unsaturated esters (**25**) with internal alkynes (**26**) with a palladium catalyst provides a variety of 3,4-disubstituted isocoumarins and polysubstituted 2-pyrones (**27**) in good yields. Formation of a seven-membered palladacyclic complex is controlled by steric factors to accomplish the regioselectivity of the reaction.

**Figure 6:**
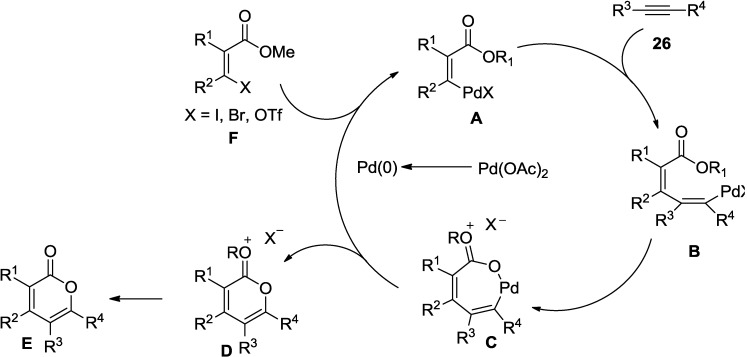
Proposed mechanism.

In view of the reaction mechanism, this annulation follows the typical palladium-catalyzed coupling sequence involving (1) reduction of Pd(II) to the actual catalyst Pd(0); (2) oxidative addition of the halide or triflate to Pd(0) (**A**); (3) vinylpalladium coordination to the alkyne and subsequent insertion to form a vinylpalladium complex (**B**); (4) formation of a seven-membered palladacyclic complex (**C**) via attack of the carbonyl oxygen on the vinylpalladium complex (**C**); and (5) reductive elimination to regenerate the Pd(0) catalyst (Figure 6).

**Scheme 9:**
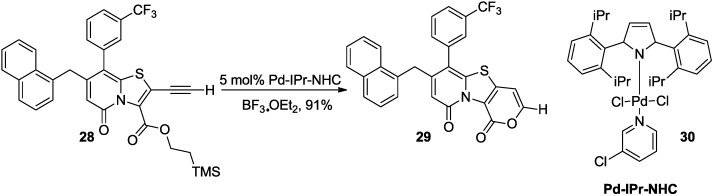
Cyclization of a carboxylic acid on an alkyne based upon *N*-heterocyclic carbenes (NHC).

In some cases, the significant problem in the synthesis of 2-pyrone via a cyclization of a carboxylic acid ester on an alkyne is the selectivity between 5-*exo*-dig and 6-*endo*-dig cyclization. In this regard, catalytic system based upon *N*-heterocyclic carbenes (NHC) developed by Almqvist and co-workers is rewarding in favor of the 6-*endo*-dig product (Scheme 9) [27]. In the reaction, selection of an appropriate Lewis acid is as much important as a catalyst. For example, addition of BF_3_.Et_2_O as a Lewis acid additive in replacement of TFA affords complete 6-*endo*-dig selectivity in the Pd-NHC catalyzed reaction.

The substituted tricyclic 2-pyrones were synthesized in outstanding yields from the corresponding internal acetylenes. This was proven to work excellently for alkyl, cycloalkyl, aryl, and heteroaryl substituted acetylenes.

**Scheme 10:**
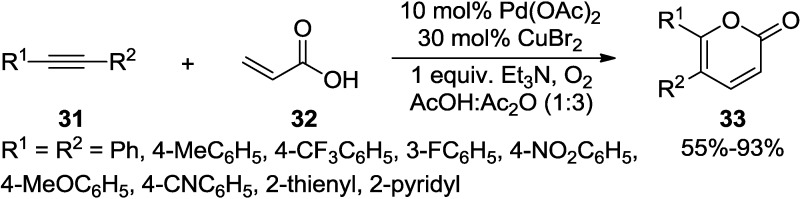
Pd-catalyzed oxidative annulations between acrylic derivatives and internal alkynes.

Jiang and co-workers reported a highly efficient strategy for the synthesis of 2-pyrones and pyridones via Pd-catalyzed oxidative annulations between acrylic derivatives and internal alkynes with high regioselectivity (Scheme 10) [28]. This process is attractive and practical because O_2_ (1 atm) is used as a stoichiometric oxidant and only H_2_O is generated as the only byproduct under mild conditions.

**Figure 7:**
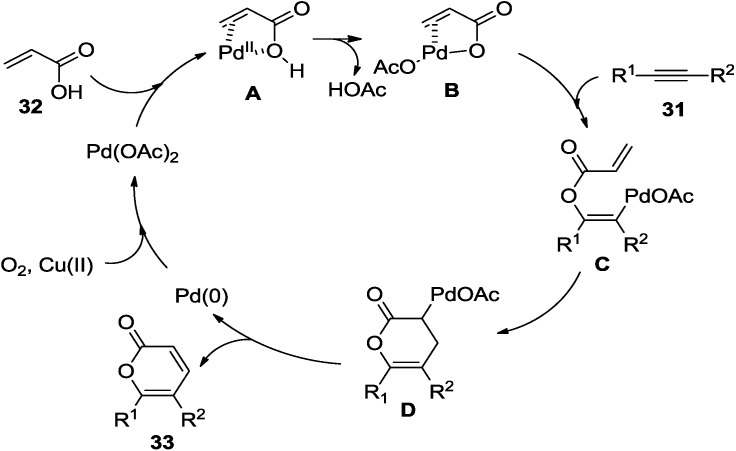
Proposed mechanism.

The coordination and ligand exchange of the acrylic derivative (**32**) with Pd(II) are involved in the initial step of the Pd-catalyzed oxidative annulation to provide X-Pd intermediate (**B**). After *exo* coordination of **31** and insertion of the diarylethyne molecule into **B** forms the vinyl-palladium complex (**C**), the intramolecular Heck-type reaction occurs to provide the alkyl-palladium species (**D**). Then, 2-pyrone (**33**) and Pd(0) would be released by β-hydride elimination and the molecular oxygen under the assistance of Cu(II) would regenerate the catalyst by oxidation of Pd(0) to Pd(II) to complete the catalytic cycle (Figure 7).

#### 2.1.2. Gold-Catalyzed Cyclo-Isomerization Strategy

**Scheme 11:**
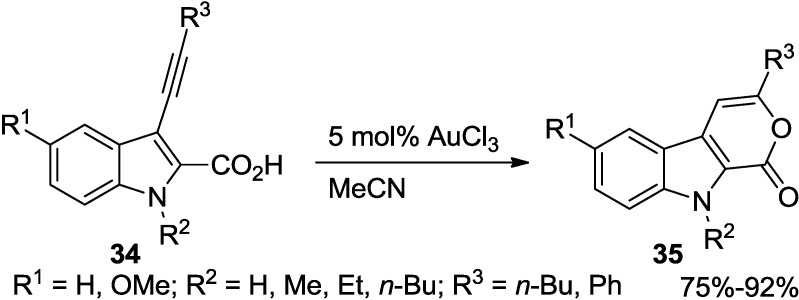
Gold-catalyzed cyclo-isomerization of 3-ethynyl-indole-2-carboxylic acid (**34**).

Due to the alkynophilic character with high functional group compatibility and many other advantages, gold catalysis has been indeed at the center of rapid development during the past decade. Recently, a highly regioselective synthesis of pyrano[3,4-b]indol-1(9*H*)-ones (**35**) via gold(III) chloride catalyzed cyclo-isomerization of 3-ethynyl-indole-2-carboxylic acid (**34**) was achieved in good to excellent yields by Perumal and co-workers (Scheme 11) [29].

In the reaction, either alkyl substitution at the nitrogen or an electron releasing substituent in the aryl ring is advantageous because this reaction proceeds at a short reaction time and provides the expected 2-pyrone in higher yields. These observations might be explained that formation of a gold complex becomes easy when the electron density of the triple bond increases.

**Scheme 12:**
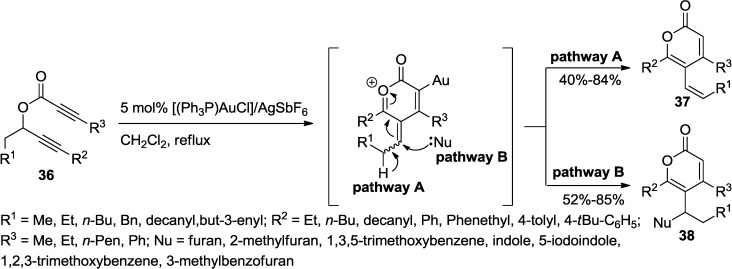
A sequential alkyne activation of allenyl propiolates by a gold(I) catalyst.

An excellent addition to this reaction category is the sequential alkyne activation of readily available allenyl propiolates by a gold(I) catalyst, [(Ph_3_P)AuCl]/AgSbF_6_ developed by the Schreiber group (Scheme 12) [30]. An intermediate oxocarbenium ion formed by a 6-*endo*-dig cyclization induced by the activation of the alkyne converts into distinct products by two pathways: H elimination or Friedel–Crafts-type addition of electron-rich aromatic and heteroaromatic derivatives (Scheme 12). In pathway A, elimination would afford a vinyl 2-pyrone (**37**) while pathway B, a Friedel–Crafts-type reaction with electron-rich aromatic and heteroaromatic compounds such as indole, furan, and benzofuran would provide a nucleophile adduct (**38**) (Scheme 12).

**Scheme 13:**
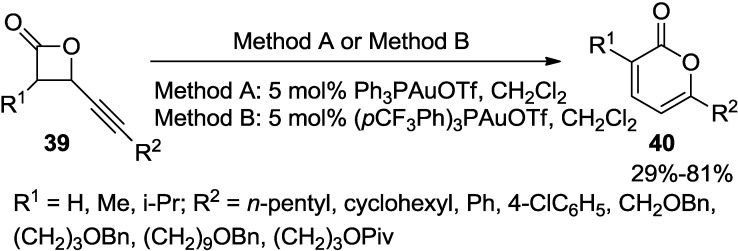
2-Pyrones from unprecedented rearrangement of β-alkynylpropiolactones (**39**).

Pale and co-workers developed a two-step procedure to substituted 2-pyrones catalyzed by a gold(I) catalyst (Scheme 13) [31]. This reaction proceeds through an unprecedented rearrangement of β-alkynylpropiolactones (**39**) to furnish 2-pyrones (**40**) in a high overall yield.

**Figure 8:**
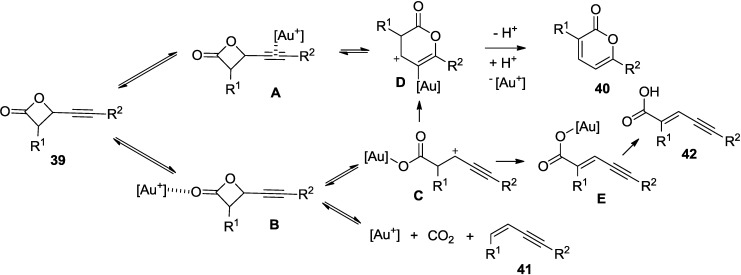
Proposed mechanism.

In a proposed mechanism, cationic pyrone gold intermediate (**D**) would be formed from both σ- and π-Au complexes (**A**) and (**B**) (Figure 8). This is possible through either a 1,3-oxygen shift or Hashmi-type cyclization from **A** and through cyclization of **C** from **B**. Elimination and subsequent protodeauration of the intermediate (**D**) would then provide the corresponding 2-pyrones (**40**). Side products, such as the enyne **41** and the acid **42**, also support σ-coordination at the β-lactone carbonyl.

In the course of a selective synthesis of the unusually sensitive cyclophanic 2-pyrone neurymenolide A (**51**), Fürstner and co-workers revisited the gold chemistry [32].

**Scheme 14:**
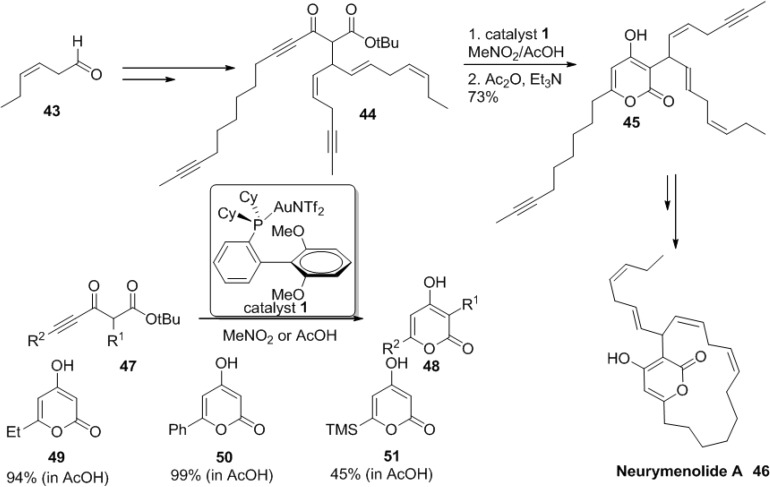
Synthesis of neurymenolide A involving a gold(I)-catalyzed 6-*endo*-dig cyclization.

With 1 mol% of [(SPhos)AuNTf_2_] in either MeNO_2_ or AcOH, ynoate (**47**) gives a smooth conversion to the corresponding 2-pyrones (**48**–**51**) (Scheme 14).

**Figure 9:**
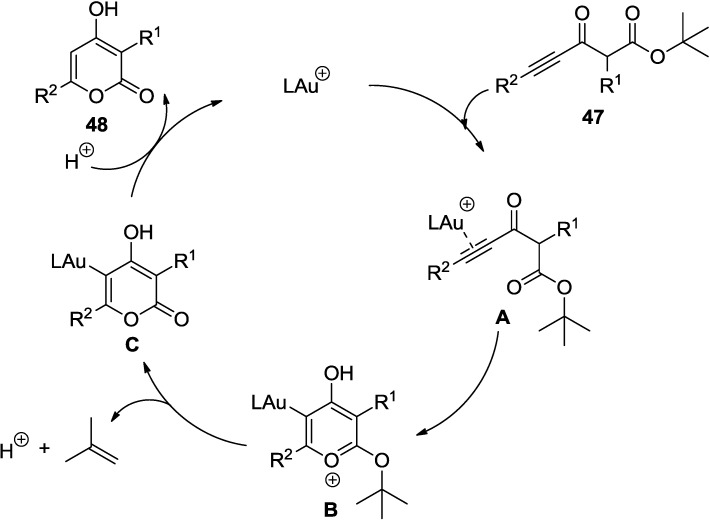
Proposed mechanism.

It is proposed that the ready cleavage of the tert-butyl group off of putative intermediate is critical for the release of the 2-pyrone ring. Later, this new method along with a ring closing alkyne metathesis (RCAM) was successfully applied to the efficient total synthesis of neurymenolide A (**46**) as key transformations (Figure 9).

**Scheme 15:**
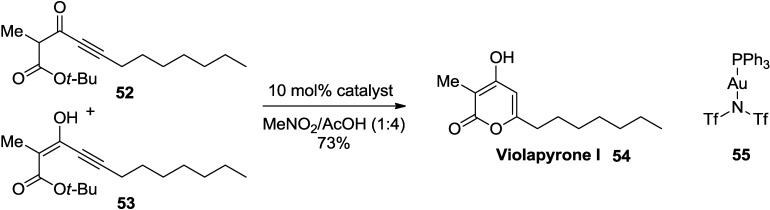
Construction of 2-pyrone ring employing a gold(I) catalyst.

Very recently, Lee and co-workers successfully applied a gold(I)-catalyzed 2-pyrone synthesis to the total synthesis of (+)-violapyrone C (Scheme 15) [33].

An inseparable mixture of β-keto ester (**52**) and its tautomer (**53**) was used as a model system for this transformation. Reaction screening with various catalysts revealed that [Bis(trifluoromethanesulfonyl)imidate](triphenylphosphine)-gold(I) in a 4:1 mixture of AcOH/MeCN at room temperature provided **54** in 73% yield.

**Scheme 16:**
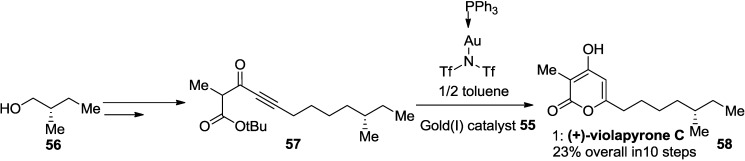
Synthesis of (+)-violapyrone C using a gold-catalyst.

Consequently, the synthesis of violapyrone C (**58**) was accomplished in 22% overall yield using the Gold(I)-catalyzed intramolecular 6-*endo*-dig cyclization of *tert*-butyl ynoates as the key reaction (Scheme 16).

**Scheme 17:**
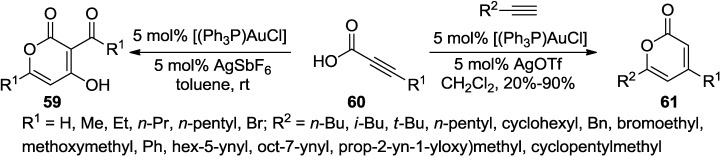
A novel cascade reaction involving propiolic acids to 2-pyrones with a gold catalyst.

Schreiber and co-workers reported that a sequential alkyne activation of terminal alkynes and propiolic acids by gold(I) catalysts furnishes 2-pyrones (Scheme 17) [34]. This novel cascade reaction involving propiolic acids give rise to 2-pyrones with different substitution patterns.

**Figure 10:**
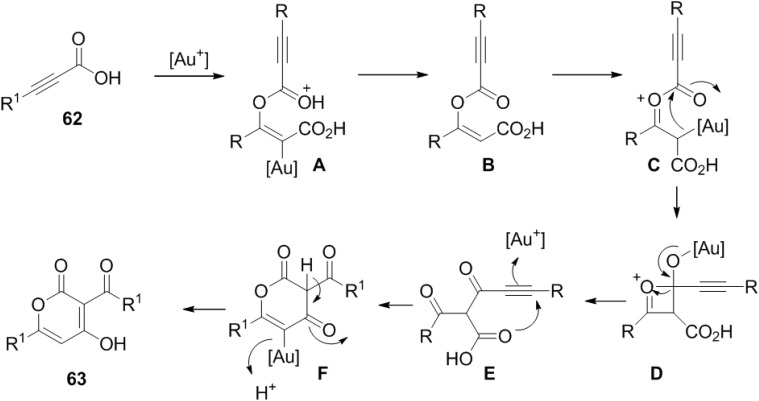
Proposed mechanism.

This reaction probably begins with addition of the carboxylic acid to the β-position of the propiolic acid (**60**) providing vinyl ester (**B**) (Figure 10). In the next step, cationic gold(I) further activates **B** to generate oxocarbenium (**C**). The acyl group is transferred to the C-Au bond of **C** to simultaneously regenerate the gold(I) catalyst. Carboxylic acid **E** initiates the 6-*endo*-dig cyclization onto the activated alkyne and enolization affords 4-hydroxy 2-pyrone (**63**).

#### 2.1.3. Rhodium-Catalyzed Synthesis of 2-Pyrones

**Scheme 18:**
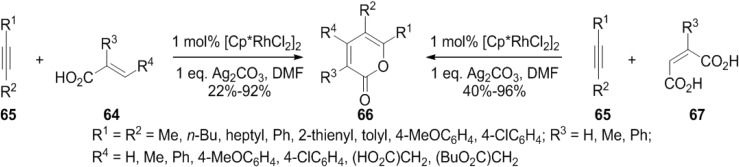
Rhodium-catalyzed oxidative coupling of substituted acrylic acids.

Miura and co-workers have achieved the straightforward and efficient synthesis of 2-pyrone and butenolide derivatives by the rhodium-catalyzed oxidative coupling reactions of substituted acrylic acids (**64**) with alkynes and alkenes (Scheme 18) [35]. This reaction proceeds via vinylic C-H bond cleavage of acrylic acids.

Later they found that the 3-unsubstituted 2-pyrones can be synthesized in good yields by using maleic acid in place of acrylic acid in their studies on the rhodium-catalyzed dehydrogenative coupling of carboxylic acids using the same catalytic system [36].

**Figure 11:**
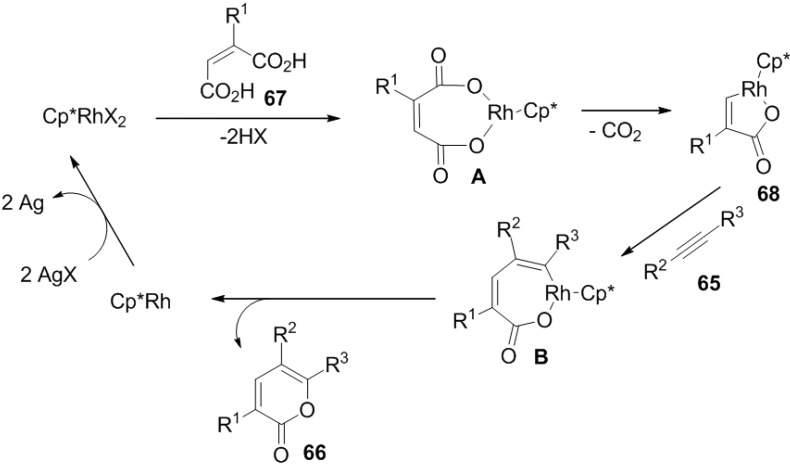
Proposed Mechanism.

Complex of **67** with a Cp*Rh(III)X_2_ species generates a rhodium(III) dicarboxylate (**A**). Subsequently decarboxylation to form a five-membered rhodacycle (**68**), alkyne insertion to give (**B**), and reductive elimination take place to produce **66**. Ag_2_CO_3_ oxidizes the resulting Cp*Rh(I) species to regenerate Cp*Rh(III)X_2_ (Figure 11).

#### 2.1.4. Ruthenium-Catalyzed Synthesis of 2-Pyrones

Almost at the same time, a similar cylization reaction was reported by Ackerman and co-workers in which [RuCl_2_(p-cymene)]_2_ was used instead [Cp*RhCl_2_]_2_ [37].

**Scheme 19:**
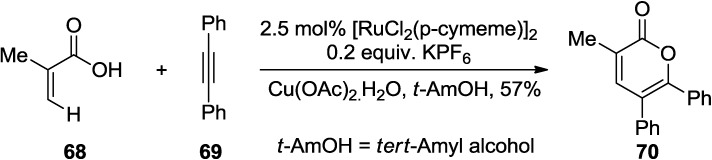
Ruthenium-catalyzed oxidative annulation of alkynes.

The cationic ruthenium(II) catalyst promotes the oxidative annulation of alkynes by acrylic acid derivative (**68**), thus providing an efficient access to 2-pyrone (**70**) (Scheme 19).

**Scheme 20:**
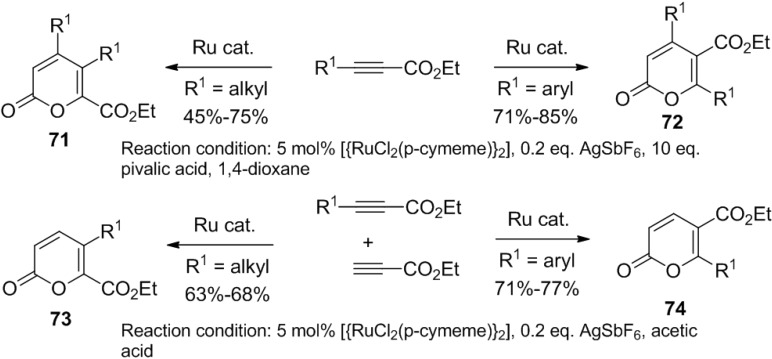
A ruthenium-catalyzed regioselective homocyclization/hetero-cross-cyclization strategy for the synthesis of 2-pyrones.

A ruthenium-catalyzed regioselective homocyclization/hetero-cross-cyclization strategy for the synthesis of 2-pyrones was developed by Jeganmohan and co-workers (Scheme 20) [38]. This reaction proceeds via alkyne/alkyne intermolecular homocyclization of substituted propiolates and hetero-cross-cyclization of substituted propiolates in the presence of a ruthenium catalyst.

Interestingly, internal alkynes prefer homocyclization, and therefore, the better choice for the reaction would be the cyclization of internal alkyne and terminal alkyne combination.

**Figure 12:**
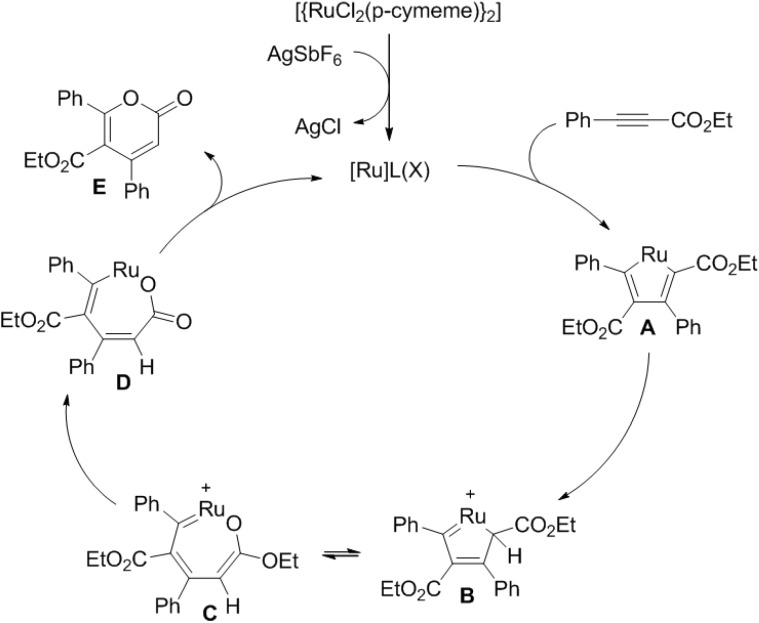
Proposed mechanism.

Removal of the chlorine ligand from the ruthenium complex by AgSbF_6_ results in a cationic ruthenium complex (Figure 12). Oxidative cyclometalation of propiolates to the complex in highly regioselective fashion generates intermediate (**A**).

Protonation only to the ester bearing carbon next to ruthenium of intermediate (**A**) by the organic acid gives intermediate (**B**). Next, nucleophilic attack of the ester group to the ruthenium in intermediate (**B**) yields intermediate (**C**). Reductive elimination of intermediate (**D**) provides cyclic product (**E**) and regenerates the ruthenium species.

**Scheme 21:**
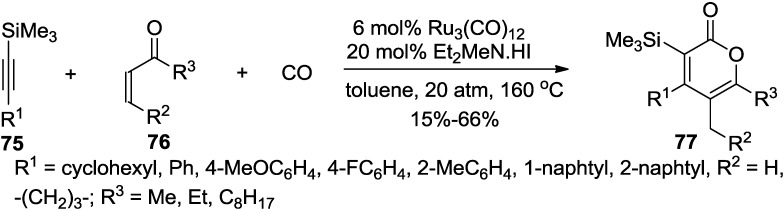
A ruthenium-catalyzed carbonylative [3 + 2 + 1] cycloaddition.

Ryu and co-workers developed a ruthenium-catalyzed carbonylative [3 + 2 + 1] cycloaddition, using silylacetylenes, α,β-unsaturated ketones, and CO to provide a new method for the synthesis of tetrasubstituted 2-pyrones (Scheme 21) [39]. In this reaction, the carbonyl group and α-carbon of vinyl ketones are combined as a three-atom assembling unit.

**Figure 13:**
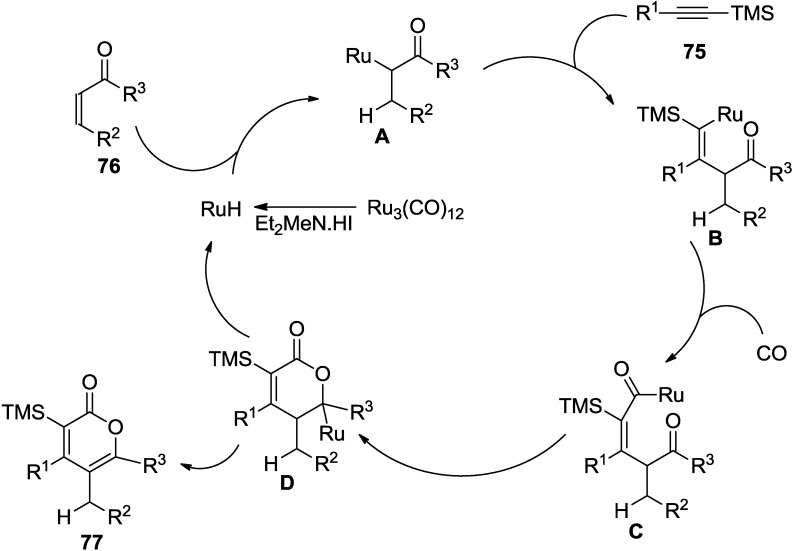
Proposed mechanism.

The ruthenium carbonyl complex generates a ruthenium hydride species with an amine-HI salt or water. This intermediate reacts with methyl vinyl ketone to give a ruthenium enolate (Figure 13). Next a vinyl ruthenium complex is generated via carboruthenation of the enolate to silylacetylene. This intermediate then undergoes CO insertion to provide an acyl ruthenium complex. Cyclization, followed by β-hydride elimination would give the 2-pyrone and regenerate the ruthenium hydride species.

#### 2.1.5. Nickel-Catalyzed Synthesis of 2-Pyrones

**Scheme 22:**
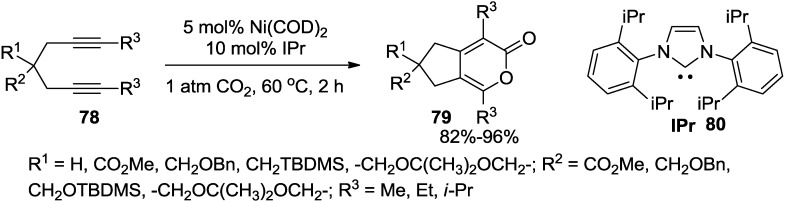
The preparation of 2-pyrones via [2 + 2 + 2] cycloaddition of diynes.

Louie and co-workers developed a mild and efficient method for the preparation of pyrones via [2 + 2 + 2] cycloaddition of diynes (**78**) and CO_2_ (Scheme 22) [40]. The reaction employs catalytic amounts of Ni(0) and IPr ligand, CO_2_(1 atm), and mild reaction conditions.

### 2.2. 2-Pyrone Synthesis Using an Organo Catalyst

**Scheme 23:**
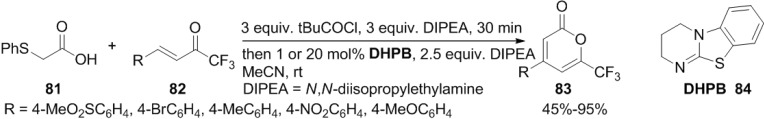
Synthesis of 2-pyrones using an organo catalyst, **DHPB**.

The synthesis of 4,6-disubstituted and 3,4,6-trisubstituted 2-pyrones from (phenylthio)acetic acids and α,β-unsaturated trifluoromethyl ketones was achieved via an one-pot isothiourea-mediated Michael addition/lactonization/thiol elimination cascade sequence (Scheme 23) [41].

**Figure 14:**
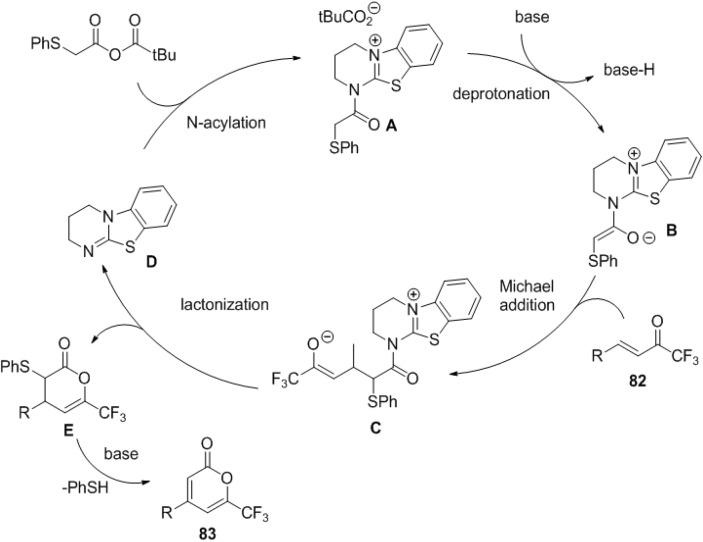
Proposed mechanism.

This transformation begins with *N*-acylation of DHPB (3,4-dihydro-2H-pyrimido[2,1-b]benzo-thiazole, **84**) with mixed anhydride (Figure 14). The (*Z*)-enolate (**B**) generated by deprotonation of **A** would undergo Michael addition to trifluoromethyl enone (**C**). Lactonization forms dihydropyrone (**E**) with concomitant regeneration of DHPB, and rapid elimination of thiophenol forms the pyrone **83**.

### 2.3. Phosphine-Catalyzed Synthesis of 2-Pyrones

**Scheme 24:**
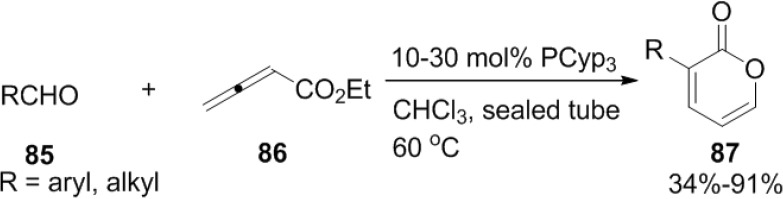
One-step phosphine-catalyzed annulation between aldehydes and ethyl allenoate.

**Figure 15:**
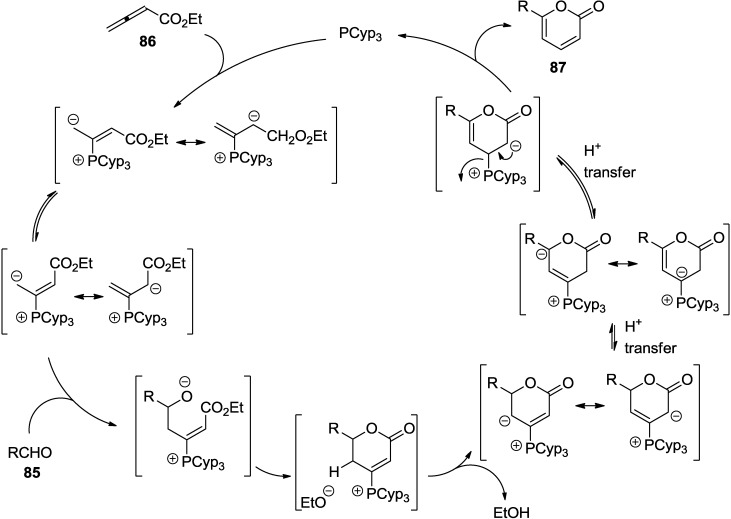
Proposed mechanism.

One-step phosphine-catalyzed annulation between aldehydes (**85**) and ethyl allenoate (**86**) to form 6-substituted 2-pyrones (**87**) was reported by Kwon and co-workers (Scheme 24) [42]. The reaction can be explained by clear discussion of the *E*/*Z*-isomerism of the zwitterion formed by the addition of a phosphine to the allenoate (Figure 15). Equilibrium shifts toward the *E*-isomeric zwitterion sterically demanding trialkylphosphines and lead to the formation of 6-substituted 2-pyrones.

### 2.4. Synthesis of 2-Pyrones via Iodolactonization

**Scheme 25:**
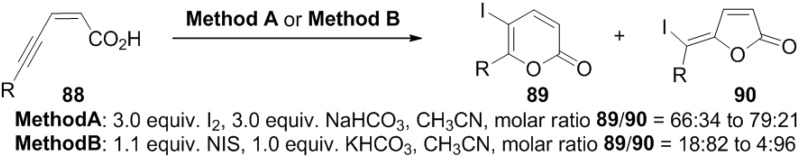
Iodolactonization strategy to 2-pyrones.

Although a selectivity problem is apparent, 2-pyrones can be prepared by iodolactonization reaction. Rossi and co-workers reported that reaction of 5-substituted (*Z*)-2-en-4-ynoic acids (**88**) with iodine and NaHCO_3_ in CH_3_CN or with ICl in CH_2_Cl_2_ affords mixtures of 6-substituted 5-iodo-2(2H)-pyranones (**89**) and (*E*)-5-(1-iodoylidene)-2(5*H*)-furanones (**90**) in which pyranones (**89**) are the major products (Scheme 25) [43].

**Scheme 26:**
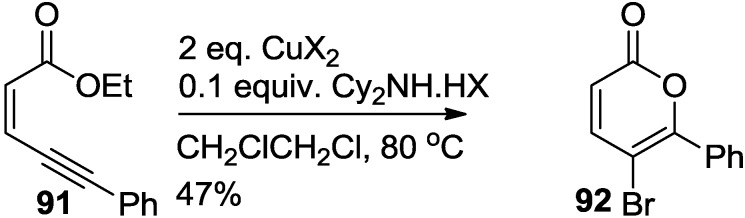
Lactonization of (*Z*)-ethyl 5-phenylpent-2-en-4-ynoate catalyzed by Cy_2_NH·HCl.

To overcome the selectivity problem existing in the iodolactonization reaction, Cy_2_NH·HX catalytic system was developed by Li and co-workers (Scheme 26) [44]. Thus, 5-bromo-2-pyrone was synthesized from the cyclization reaction of (*Z*)-pent-2-en-4-ynoate with CuBr_2_. When 0.1 equivalent of Cy_2_NH·HCl was added in combination with two equivalents of CuBr_2_, cyclization of (*Z*)-ethyl 5-phenylpent-2-en-4-ynoate underwent a smooth conversion to the corresponding product in 47% in 6 h. This reaction extends to a variety of *o*-(alk-1-ynyl)benzoates to provide the corresponding 4-haloisocoumarins in moderate to excellent yields.

### 2.5. Synthesis of 2-Pyrones via Baylis-Hillman Reaction

**Scheme 27:**
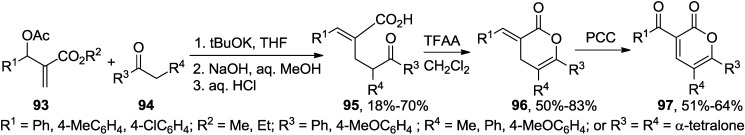
Application of Baylis–Hillman reaction to the synthesis of 2-pyrone.

Baylis–Hillman adducts transformed to 3,5,6-trisubstituted 2-pyrones (**97**) (Scheme 27). The synthesis was carried out via the sequential introduction of ketone at the primary position of Baylis–Hillman adduct, lactonization, and the oxidation with PCC [45].

### 2.6. Miscellaneous

#### 2.6.1. Ring Expansion Strategy

**Scheme 28:**
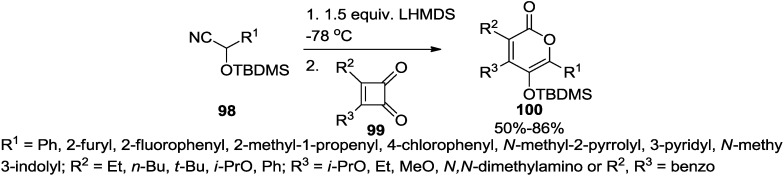
Synthesis of 2-pyrones employing a lithiated *O*-silylated cyanohydrin.

Liebeskind and co-workers found that the addition of a lithiated *O*-silylated cyanohydrin to a cyclobutenedione (**99**) with subsequent intramolecular 1,4-silyl migration and displacement of cyanide generates *in situ* a 4-acylcyclobutenone, which undergoes spontaneous ring expansion to a substituted 2-pyrone (**100**) in good to excellent yield (Scheme 28) [46].

**Figure 16:**
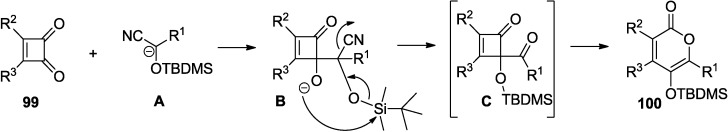
2-Pyrone synthesis involving a ring expansion.

The facile ring expansion of the 4-acylcyclobutenone at or below room temperature is a unique feature of this reaction since heating at temperatures in excess of 100 °C is required in most ring expansions of 4-aryl- or 4-vinylcyclobutenones (Figure 16).

**Scheme 29:**
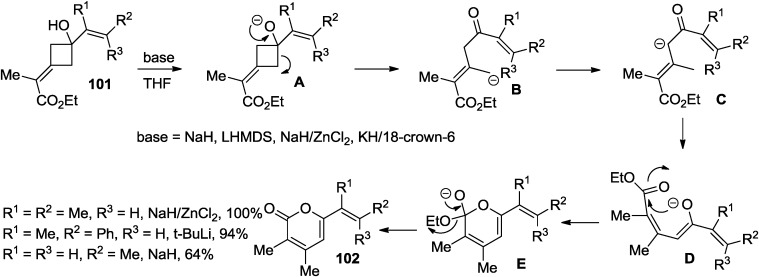
A ring opening of the metal alkoxide (**A**) to 2-pyrones.

Substituted 3,4-dimethyl-2-pyrones (**102**) were prepared in good yields by treatment of 3-carboethoxyethylidene cyclobutanols with various bases (Scheme 29) [47]. Jung and co-workers proposed a mechanism involving ring opening of the metal alkoxide (**A**) to give the carbanion (**B**), which undergoes proton transfer to give the more stable carbanion (**C**) and double bond isomerization to give the enolate (**D**) to form the pyrone ring (**102**) via attack on the ester.

#### 2.6.2. Condensation

**Scheme 30:**
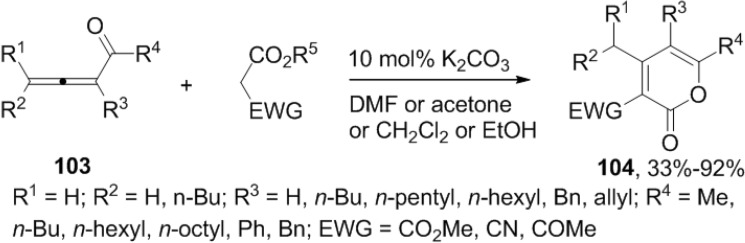
A base-catalyzed or promoted reaction of 1,2-allenyl ketones (**103**) with electron-withdrawing group substituted acetates.

**Figure 17:**
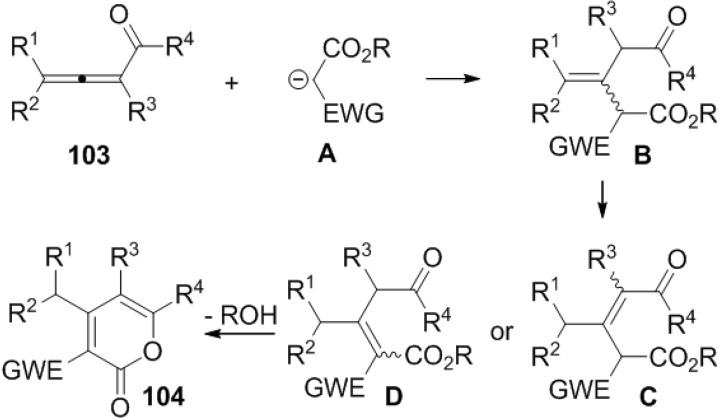
Proposed mechanism.

A base catalyzed or promoted reaction of 1,2-allenyl ketones (**103**) and electron-withdrawing group substituted acetates provides 2-pyrone derivatives (Scheme 30). The reaction proceeds through a Michael addition C-C double-bond migration−lactonization process (Figure 17) [48,49].

**Scheme 31:**
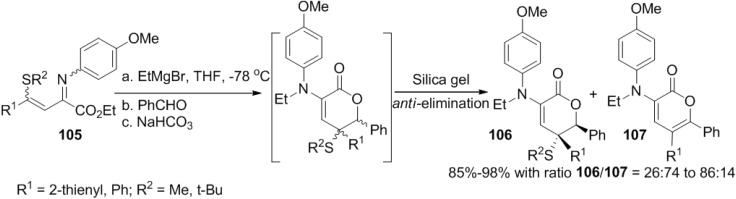
Application of tandem *N*-alkylation/vinylogous aldol reaction of β,γ-alkenyl α-iminoesters (**105**) to the synthesis of 2-pyrone.

Shimizu and co-workers described a highly regioselective tandem *N*-alkylation/vinylogous aldol reaction of β,γ-alkenyl α-iminoesters (**105**) (Scheme 31) [50]. In this reaction, the sulfide group enhances the regioselectivity of the directed vinylogous aldol reaction to afford a new synthetic method of 3-amino-2-pyrones.

Although δ-lactone (**106**) was the major component of the crude products, this was converted into 2-pyrone (**107**) in silica gel TLC chromatography purification.

**Figure 18:**
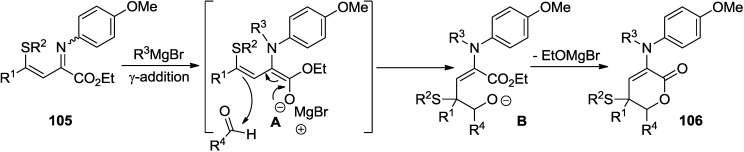
Proposed mechanism.

β,γ-Alkenyl α-iminoesters (**105**) exist as a mixture of (*Z*)- and (*E*)-diastereomers. Heating above room temperature promotes the isomerization of the inert (*Z*)-diastereomers into the reactive (*E*)-diastereomers (Figure 18). *N*-alkylation of the (*E*)-diastereomers with the Grignard reagent provides magnesium dienolide (**A**) via a five-membered intermediate comprising the imino nitrogen, the carbonyl oxygen, and the magnesium atom. γ-Addition of dienolide (**A**) to the aldehyde forms magnesium aldolate (**B**), followed by a concomitant intramolecular cyclization to furnish δ-lactone (**106**).

**Scheme 32:**
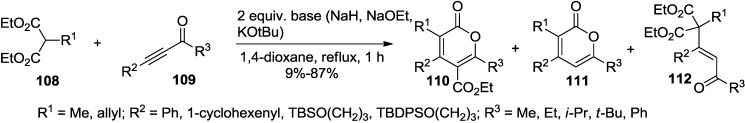
1,4-Addition of active methane compounds to 2-alkynone (**109**) for the synthesis of 2-pyrone.

The 1,4-addition of active methylene compounds to 2-alkynone (**109**) for the synthesis of 2-pyrone was reported by Shimizu and co-workers (Scheme 32) [51]. The reactions provide 3,4,6-trialkyl-5-ethoxycarbonyl or 5-acetyl-2-pyrones via a novel reaction mechanism involving cyclobutenoxide intermediates.

**Scheme 33:**
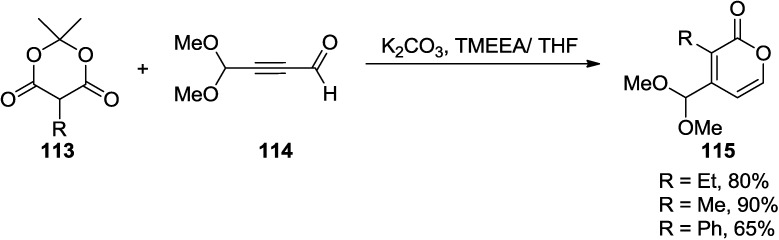
A reaction of acetylene dicarboxaldehyde monoacetal (**114**) with substituted Meldrum’s acid (**113**).

Rigo and co-workers found a reaction of acetylene dicarboxaldehyde monoacetal (**114**) with substituted Meldrum’s acid leads to 2-pyrone-4-carboxaldehydes (**115**) in good yields (Scheme 33) [52].

**Scheme 34:**
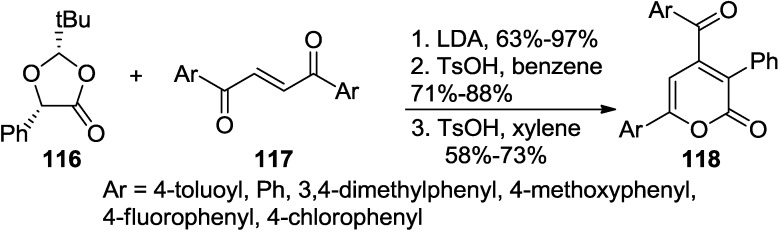
A three-step procedure of the Michael addition, cyclization, and lactonization to 2-pyrone.

**Figure 19:**
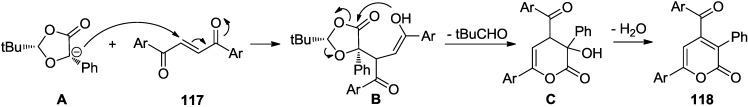
Proposed mechanism.

A three-step procedure of the Michael addition, cyclization to a furan, and lactonization provided 4-aroyl-6-aryl-3-phenyl-2-pyrones (**118**) (Scheme 34, Figure 19) [53]. Under prolonged or strong acidic treatment, the Michael products directly converted to these 2-pyrones with little decrease in yield.

**Scheme 35:**
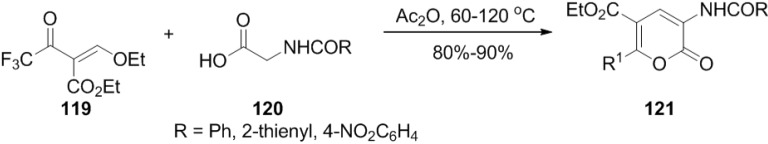
Synthesis of 2-pyrone from a reaction of β-alkoxyvinyl polyfluoroalkyl ketones (**119**) with *N*-acylglycines.

Condensation of β-alkoxyvinyl polyfluoroalkyl ketones (**119**) with *N*-acylglycines (**120**) in acetic anhydride provided a number of 3-acylamino-6-polyfluoroalkyl-2H-pyran-2-ones (**121**) in high yield (Scheme 35) [54].

**Scheme 36:**
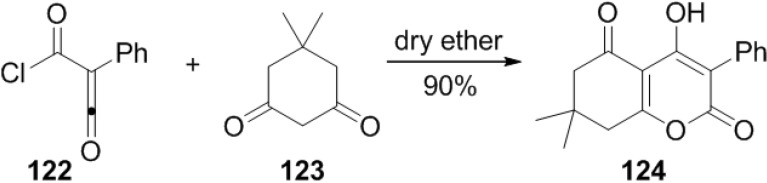
A reaction of (chlorocarbonyl)phenyl ketene (**122**) with various 1,3-diketones.

A reaction of (chlorocarbonyl)phenyl ketene (**122**) with various readily available 1,3-diketones afforded 2-pyrone derivatives (**124**) in an one step procedure (Scheme 36). This method provides an easy way to synthesize 3,4,5,6-tetrasubstituted 2-pyrones in good to excellent yields [55].

**Figure 20:**
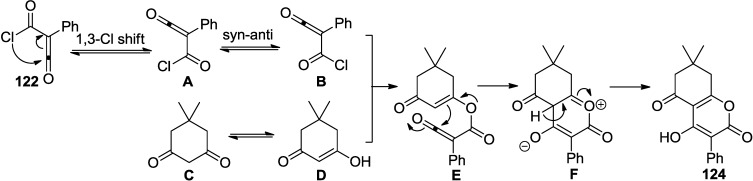
Proposed mechanism.

It is of note that ketene (**122**) undergoes 1,3-shift of chlorine, which was determined by ^13^C NMR experiment (Figure 20). Thus, the chlorine atom interchanges between two carbonyl groups and also these ketenes can exist as a mixture of two conformers, such as *s-cis* (**A**) and *s-trans* (**B**). The reaction completed by nucleophilic attack of the OH group of the enol form (**D**) at the acyl carbonyl position of the ketene, followed by ring closure.

**Scheme 37:**
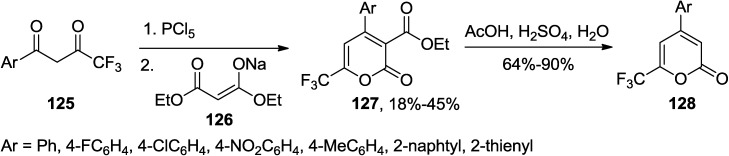
A reaction of aryl-4,4,4-trifluorobutane-1,3-diones and sodium diethyl malonate.

Usachev and co-workers developed a convenient synthesis of a series of 2-pyrones with a CF_3_ group at the 6-position and aryl group at position 4, from readily available aryl-4,4,4-trifluorobutane-1,3-diones (**125**), PCl_5_, and sodium diethyl malonate (**126**) (Scheme 37) [56].

#### 2.6.3. Rearrangement-Cyclization Strategy

**Scheme 38:**
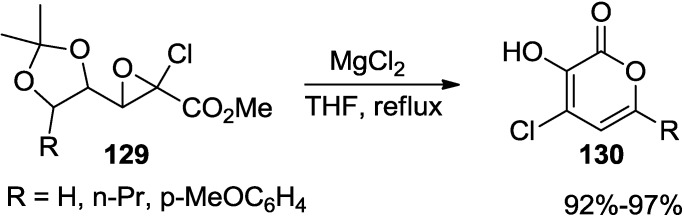
One pot rearrangement-cyclization reaction with MgCl_2_.

Tsuboi and co-workers reported that a novel one pot rearrangement–cyclization reaction of acetonide protected 4,5-dihydroxy-2-chloroglycidic ester (**129**) or its rearrangement product with magnesium halides provided 4-halo-3-hydroxy-2-pyrones (**130**) in excellent to reasonable yields in one pot (Scheme 38) [57].

**Figure 21:**
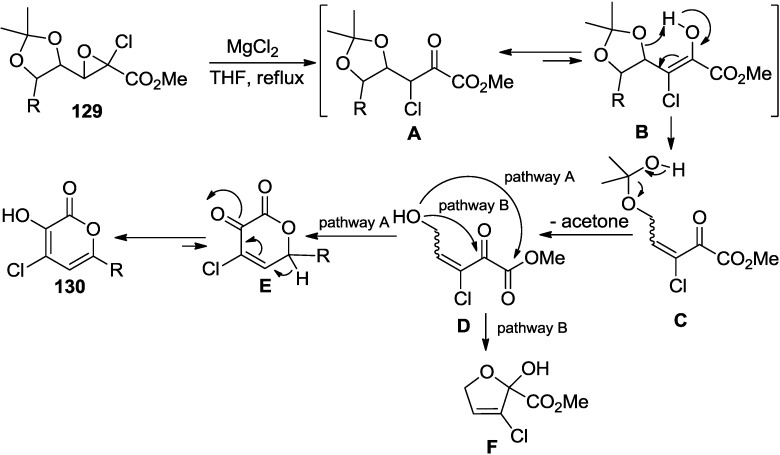
Proposed mechanism.

The experimentally proven mechanism describes that the reaction is initiated by the magnesium chloride-mediated conversion of glycidic ester (**129**) to keto ester (**A**) (Figure 21). Subsequently, elimination of acetone via keto-enol tautomerism results in formation of intermediate **D**. At this stage, the reaction proceeds in two different plausible pathways. For example, nucleophilic addition of hydroxyl group to ester carbonyl carbon (pathway A) would provide 4-chloro-3-hydroxy-2-pyrone (**130**) after elimination of MeOH. In contrast, nucleophilic attack at keto carbonyl position (pathway B) would furnish 3-chloro-2-hydroxy-2,5-dihydrofuran-2-carboxylate (**F**).

**Scheme 39:**
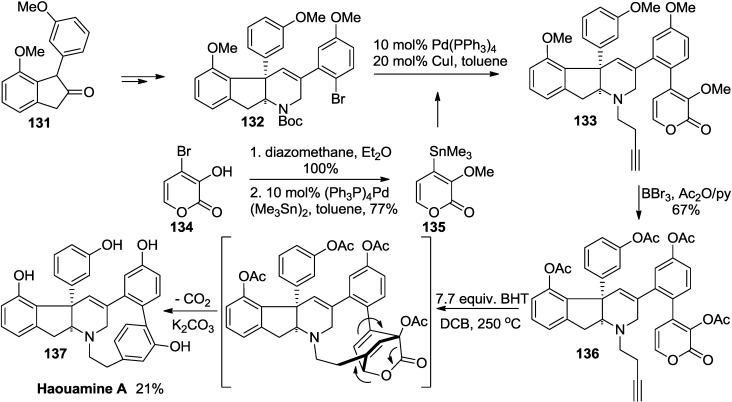
Total synthesis of haouamine A (**137**).

Later, Baran and co-workers applied this method to the total synthesis of (±)-haouamine A (**137**) on the premise that a cyclohexadiene from the pyrone-alkyne Diels-Alder reaction can serve as a viable precursor since it can release CO_2_ to undergo subsequent aromatization (Scheme 39) [58].

In consequence, the total synthesis of the structurally complex haouamine A (**137**) was realized in eight steps from readily available indanone (**131**) via the unprecedented chemistry specifically tailored for a pyrone-assisted stitching of the bent aromatic ring.

**Scheme 40:**
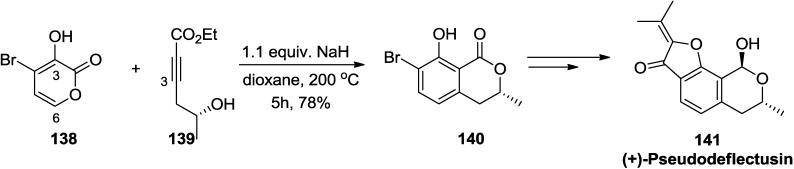
Synthesis of (+)-pseudodeflectusin (**141**).

As another example, the second-generation synthesis of (+)-pseudodeflectusin (**141**) was accomplished in five steps, featuring a base-facilitated Diels-Alder reaction between 2-pyrone (**138**) and alkyne (**139**), lactonization, and decarboxylation (Scheme 40) [59]. Especially, it is of note that enhancement of the HOMO coefficient at C-6 induced by deprotonation of the 3-OH resulted in the reaction of the C-3 of alkyne (**139**) exclusively with the C-6 of 2-pyrone (**138**) under basic conditions to furnish the desired regioisomer **140**.

## 3. Synthetic Application of Substituted 2-Pyrones

### 3.1. Synthetic Application of 3,5-Dibromo-2-Pyrone

Due to the outstanding stereochemical outcome in the Diels-Alder cycloadditions, substituted 2-pyrones have been extensively used as key intermediates in the synthesis of complex natural products. Especially, brominated 2-pyrones are attractive ambident dienes as they can react with both electron poor and rich dienophiles via normal- and inverse-electron-demand Diels-Alder cycloadditions with good stereocontrol. The dual reactivity of brominated 2-pyrones is resulted from the fact that the bromine atom at 3- or 5-position on the 2-pyrone ring can either withdraw or donate electron density to the diene moiety of the 2-pyrone ring. However, despite the versatility and usefulness of brominated 2-pyrones, their synthetic application has been hampered by the limited accessibility.

**Scheme 41:**
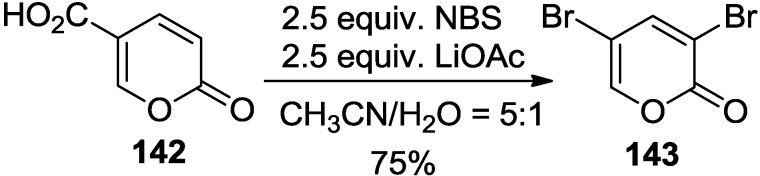
Synthesis of 3,5-dibromo-2-pyrone (**143**).

Recently, this problem has been ameliorated by Cho and co-workers. Thus, bromo-decarboxylation of 2-pyrone-carboxylic acids under the condition with NBS (1.2~2.5 equiv.), LiOAc (1.2~2.5 equiv.) in aqueous CH_3_CN furnished various brominated 2-pyrones in good to fair yields [60]. It is of note that 3,5-dibromo-2-pyrone (**143**), an ambiphilic diene, was obtained in 75% isolated yield when 2.5 equiv. of both NBS and LiOAc were used (Scheme 41).

**Scheme 42:**
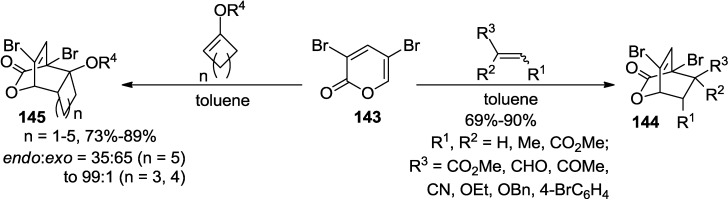
Diels-Alder reaction of 3,5-dibromo-2-pyrone.

Having a practical method for the synthesis of 3,5-dibromo-2-pyrone, [4 + 2] Diels-Alder cycloadditions were examined with a series of electronically and sterically distinct dienophiles, including various electron-poor and -rich dienophiles (**144**, Scheme 42) [60,61,62], even highly hindered cycloalkenyl enol ethers (**145**, Scheme 42) to provide tricyclolactones with good to excellent chemical yields and *endo*/*exo* regioselectivity [63]. Consequently, its high potency as an ambident diene resulted in a higher reactivity and a better stereoselectivity compared to monobromo-2-pyrones, thus generating a variety of bicycloadducts in much higher chemical yields (**144**, Scheme 42). The results of these experiments led to a conclusion that the bromo-2-pyrone series have an order of reactivity: 3,5-dibromo-2-pyrone > 5-bromo-2-pyrone > 3-bromo-2-pyrone.

Later, the investigation further extended to cycloadditions with highly sterically hindered cycloalkenyl enol ethers to provide a series of tricyclolactones, which was unprecedented in the past (**145**, Scheme 42) [63]. With regard to the stereochemical outcome of the cycloadditions, mostly *endo*-products were prepared with cyclic enol ethers up to seven-membered ring systems while *exo*-product was produced in moderate selectivity.

**Scheme 43:**
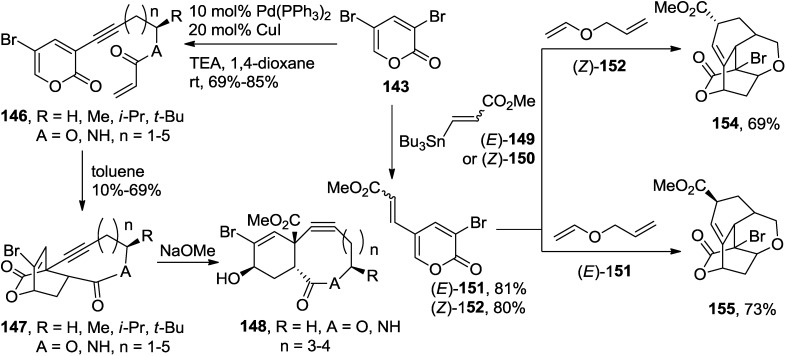
Intramolecular, tandem Diels-Alder reactions of 3,5-dibromo-2-pyrone.

On the other hand, 2-pyrones attached with acrylate or acrylamide dienophiles through alkynyl tethers, which are synthetically accessible from the Sonogashira coupling reactions of 3,5-dibromo-2-pyrone, undergo Type-I intramolecular Diels-Alder (IMDA) reactions to provide bicyclolactone-fused 9- to 11-membered macrolactones (**147**) with high diastereofacial and *endo*-selectivity (Scheme 43) [64,65]. Interestingly, the diastereofacial selectivity of the IMDA reactions decreases as the size of the alkyl group adjacent to the macrocyclic ester oxygen increases (**146**, R = *i*-Pr, A = O, *n* = 3 *vs.* **146**, R = *t*-Bu, A = O, *n* = 3, Scheme 43) [65]. This result can be explained that the bulkier substituent would exert an increased level of steric repulsion, diminishing the energy difference between two corresponding transition states. The ring size of the resulting macrocycles is also important (**146**, R = Me, A = O, *n* = 1 *vs.* **146**, R = Me, A = O, *n* = 5, Scheme 43) [65]. Therefore, in general, smaller macrocycles have the higher observed diastereofacial selectivity.

3-Bromo-2-pyrone-5-carboxylates ((*E*)-**151**, (*Z*)-**152**), readily prepared from the regioselective Pd-catalyzed coupling reactions of 3,5-dibromo-2-pyrone (**143**), were converted into the corresponding tetracyclolactones via tandem sequential Diels-Alder cycloaddition reactions in high regio- and stereoselectivity (**154**, **155**, Scheme 43) [66]. In the reaction sequence, the incorporation of a vinyl group at the C5 position of 3,5-dibromo-2-pyrone (**143**), prior to the intermolecular cycloaddition with an allyl vinyl ether-type bisdienophile, allows direct delivery of the polycarbocycles in a single operation (Scheme 43).

**Scheme 44:**
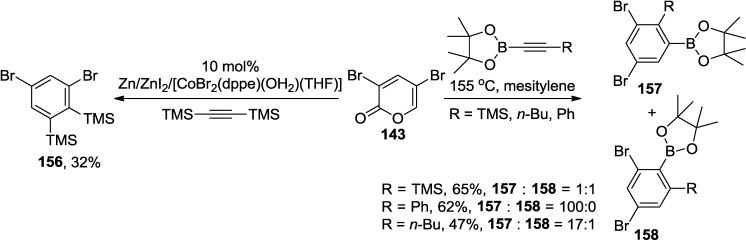
Cycloadditions of 3,5-dibromo-2-pyrone with substituted acetylenes.

The cycloadditions of 3,5-dibromo-2-pyrone (**143**) with substituted acetylenes including alkynylboronates and 1,2-bis(trimethylsilyl)acetylene (BTMSA) deliver a series of functionalized aromatic compounds without any further treatment (Scheme 44) [67,68]. It is of note that the selectivity is modulated by substituents on the alkynes. Therefore, an excellent regioselectivity was observed with phenyl- or *n*-Bu substituted boronates whereas an equal mixture of regioisomers was generated with the trimethylsilyl substituted acetylene (Scheme 44) [67].

**Scheme 45:**
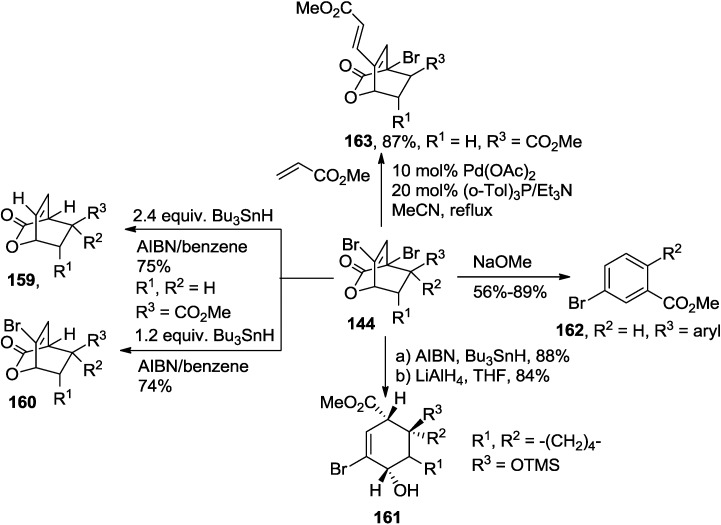
Further transformations of the cycloadduct (**144**).

The resulting cycloadducts (**144**) from the previously described Diels-Alder cycloaddition reactions were further transformed into corresponding bicarbocyclic skeletons or aromatic compounds (Scheme 45). For example, the two bromine groups on the resulting cycloadducts can be independently manipulated to produce more complex bicyclolactones (**159**, **160**, Scheme 45). Thus, selective debromination under typical Bu_3_SnH/AIBN condition and subsequent reductive lactone ring opening reaction with LAH provided the corresponding ester (**161**), and the direct opening of the lactone bridge with NaOMe was accompanied with a concomitant 1,4-elimination and subsequent aromatization to provide the aromatic compounds (**162**, Scheme 45) [61].

Another synthetically useful feature of the cycloadducts (**143**) is that the Heck coupling reaction with methyl acrylate gave rise to the dienoate (**163**) in 87% (Scheme 45) [61].

**Scheme 46:**
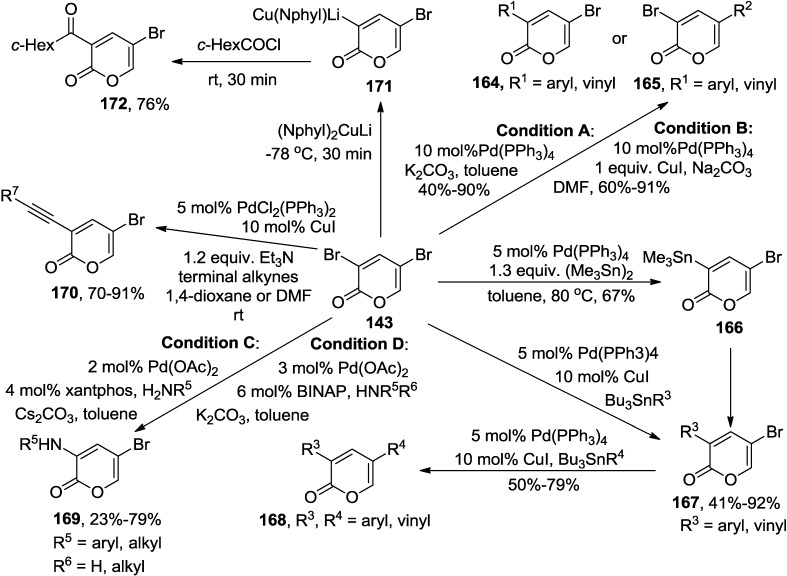
Metal-catalyzed coupling reactions of 3,5-dibromo-2-pyrone (**143**).

Cho and co-workers have examined various transition metal catalyzed coupling reactions with 3,5-dibromo-2-pyrone (**143**), thereby broadening the scope of the reactions. Consequently, it was observed that the 2,5-dibromo-2-pyrone (**143**) undergoes Pd-catalyzed regioselective Suzuki-Miyaura coupling, stannylation, Stille coupling, amination, and Sonogashira alkynylation (Scheme 46).

Suzuki-Miyaura coupling reactions of 2,5-dibromo-2-pyrone (**143**) with aryl- and vinyl-boronic acids provided either 3- (**Condition A**) or 5-substituted 2-pyrones (**Condition B**) in high regioselectivity, depending upon the reaction conditions [69]. Interestingly, solvent polarity and the existence of Cu(I) play a decisive role in the regioselectivity of the coupling reactions. Although 3-(trimethylstannyl)-5-bromo-2-pyrone (**166**) reacted further with various aryl halides under the Stille coupling condition to afford a series of 3-aryl-5-bromo-2-pyrones (**167**) with high regioselectivity, this approach suffers from synthetic inconvenience as it requires a separate preparation of 3-stannylated-2-pyrone, and furthermore, it is not effective in the coupling reaction as well [70,71]. This shortcoming was overcome by a direct coupling of 3,5-dibromo-2-pyrone (**143**) with various aryl, heteroaryl and vinyl stannanes to furnish diverse 3-substituted 5-bromo-2-pyrones (**167**, Scheme 46) [72]. It is of note that Stille coupling reactions occur regioselectively at either C3 (Pd(PPh_3_)_4_/10 mol% CuI/toluene) or C5 (Pd(PPh_3_)_4_/1 equiv. CuI/DMF), depending on the reaction conditions. Subsequent second coupling reaction of the corresponding 3-substituted-5-bromo-2-pyrones (**167**) provided a series of structurally interesting 2-pyrones (**168**), bearing two different groups in 50%–79% yields. As another intriguing example, a variety of previously unknown 3-arylamino- and 3-alkylamino-5-bromo-2-pyrones (**169**) were prepared by the Pd-catalyzed regioselective amination reactions (Scheme 46) [73]. Various aryl amines underwent coupling reactions to afford 3-arylamino-5-bromo-2-pyrones in fair to good yields (**Condition C**, Scheme 46). In contrast, the amination with alkyl amines required a different reaction condition to provide 3-alkylamino-5-bromo-2-pyrones (**Condition D**, Scheme 46). It is noteworthy that the lower nucleophilicity of secondary amines resulted in improved yields.

Additionally, regioselective Sonogashira alkynylations at the C3 position of 3,5-dibromo-2-pyrone (**143**) were established with terminal alkynes to afford various synthetically useful 3-alkynyl-5-bromo-2-pyrones (**170**) [74]. Besides above-mentioned coupling reactions, Knochel and co-workers investigated a bromide-copper exchange by the reaction of 3,5-dibromo-2-pyrone (**143**) with a sterically hindered cuprate reagent, lithium dineophylcuprate (Me_2_PhCCH_2_)_2_CuLi, leading to the copper derivative (**171**) which can be readily acylated with cyclohexanecarbonyl chloride to provide the ketone (**172**) in 76% yield (Scheme 46) [75].

Based on the cumulative information on the reactivity of 3,5-dibromo-2-pyrone (**143**), several excellent total syntheses of complex natural products including (±)-*trans*-dihydronarciclasine [76,77], (±)-crinine [78,79], (±)-crinamine [78], (±)-pancratistatin [80,81], (±)-galanthamine [82], (±)-aspidospermidine [83], (±)-joubertinamine [84], (±)-lycorane [85,86], *etc.*, have been accomplished.

### 3.2. Synthetic Application of 4-Bromo-6-Methyl-2-Pyrone

4-Bromo-6-methyl-2-pyrone (**173**) is an attractive class of 2-pyrone family, hosting a broad range of reactions to maximize its synthetic utility. It is easily accessible from bromination of commercially available 4-hyroxy-6-methyl-2-pyrone using PCl_3_ or TBAB (tetrabutylammonium bromide) via Vilsmeier Haack type reaction [87]. The 4-carbon center is very reactive towards oxidative addition to Pd(0) species as it is an electron deficient carbon within the 2-pyron e ring and therefore an activated position.

**Scheme 47:**
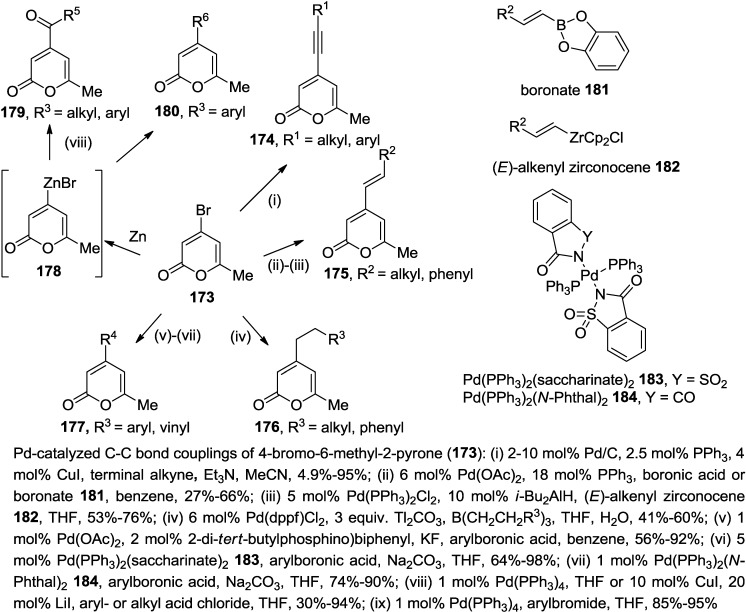
Synthesis of 4-substituted 2-pyrones.

Many biologically active 4-alkynyl- and 4-alkenyl-6-methyl-2-pyrones were efficiently synthesized using Pd-catalyzed coupling reactions (Scheme 47). For example, various 4-alkynyl-6-methyl-2-pyrones (**174**) were prepared under the condition of Pd/C with added Ph_3_P as the catalyst in the presence of catalytic CuI in a mixture of MeCN and Et_3_N through Sonogashira cross-coupling reactions of 4-bromo-6-methyl-2-pyrone (**173**) [88,89,90,91,92,93]. The concentration of Pd is also of great importance to the product yield as lower Pd-loadings favor higher conversions and purer cross-coupled product.

The synthesis of 4-alkenyl-6-methyl-2-pyrones (**175**) was accomplished by Suzuki-Miyaura coupling reactions with alkenylboronic acids or alkenylboronates (**181**) in the prescence of Pd(OAc)_2_ or Pd(PPh_3_)_2_(*N*-Phthal)_2_ [88,89,91,94]. On the other hand, Negishi coupling reactions of 4-bromo-6-methyl-2-pyrone (**173**) with *E*-alkenyl zirconocenes (**182**) provided the same products in slightly improved yields [91]. Interestingly, only the *E*-isomer was detected in these reactions, showing that the geometry of the zirconocenes (**182**) is retained during the reactions.

Other than the alkenylation of 4-bromo-6-methyl-2-pyrone, Suzuki-Miyaura coupling reaction was applied further to the syntheses of 4-alkyl- and 4-aryl-6-methyl-2-pyrones (**176**, **177**, Scheme 47). Therefore, a number of readily accessible trialkylboranes were coupled with 4-bromo-6-methyl-2-pyrone (**173**) under the condition of Pd(dppf)Cl_2_ (dppf: 1,1′-bis(diphenylphosphino)ferrocene) and Tl_2_CO_3_ [91]. The advantages of using the dppf ligand over others are that it circumvents β-hydride elimination of the alkyl-Pd intermediates, reduction of the halide and isomerization of the alkyl groups and, therefore, significantly enhances the efficient transfer of the alkyl group to the 2-pyrone. With regard to the arylation of 4-bromo-6-methyl-2-pyrone (**173**) via Suzuki-Miyaura coupling reactions, several conditions using Pd(OAc)_2_ [89,91], Pd(PPh_3_)_2_(saccharinate)_2_ (**183**) [95], and Pd(PPh_3_)_2_(*N*-Phthal)_2_ (**184**) [94] have been developed to provide the corresponding 4-aryl-6-methyl-2-pyrones (**177**). Additionally, 4-pyronylzinc bromide, which is prepared by the reaction of 4-bromo-6-methyl-2-pyrone (**173**) with activated zinc undergoes coupling reactions with aryl acid chlorides and aryl halides to afford the corresponding ketones (**179**) and 4-aryl-6-methyl-2-pyrones (**180**), respectively [96].

## 4. Conclusions

Over the past two decades, we have witnessed great strides in our understanding of 2-pyrone chemistry. In a synthetic point of view, the development of new methodology for the efficient synthesis of 2-pyrones has been at the center of much attention in the field of organic synthesis. As a result, a range of synthetic methodologies have been developed so far and they are categorized into several classes, including (1) metal-catalyzed synthesis of 2-pyrones; (2) synthesis of 2-pyrones using an organo catalyst; (3) phosphine-catalyzed synthesis of 2-pyrones; (4) synthesis of 2-pyrones via iodolactonization; (5) synthesis of 2-pyrones via Baylis-Hillman reaction; (6) synthesis of 2-pyrones via ring expansion strategy; (7) synthesis of 2-pyrones via base-promoted condensation; (8) synthesis of 2-pyrones via rearrangement-cyclization strategy, *etc.* Additionally, the chemistry of 3,5-dibromo-2-pyrone (**143**) and 4-bromo-6-methyl-2-pyrone (**173**) has been well established by the pioneering works of Cho and Fairlamb and it is currently at the stage to provide useful tools for the synthesis of complex natural products.
